# Stress induced *Salmonella *Typhimurium recrudescence in pigs coincides with cortisol induced increased intracellular proliferation in macrophages

**DOI:** 10.1186/1297-9716-42-118

**Published:** 2011-12-07

**Authors:** Elin Verbrugghe, Filip Boyen, Alexander Van Parys, Kim Van Deun, Siska Croubels, Arthur Thompson, Neil Shearer, Bregje Leyman, Freddy Haesebrouck, Frank Pasmans

**Affiliations:** 1 Department of Pathology, Bacteriology and Avian Diseases, Faculty of Veterinary Medicine, Ghent University, Salisburylaan 133, 9820 Merelbeke, Belgium; 2 Department of Pharmacology, Toxicology and Biochemistry, Faculty of Veterinary Medicine, Ghent University, Salisburylaan 133, 9820 Merelbeke, Belgium; 3 Department of Foodborne Bacterial Pathogens, Institute of Food Research, Norwich Research Park, Colney Lane, Norwich NR4, UK

## Abstract

*Salmonella *Typhimurium infections in pigs often result in the development of carriers that intermittently excrete *Salmonella *in very low numbers. During periods of stress, for example transport to the slaughterhouse, recrudescence of *Salmonella *may occur, but the mechanism of this stress related recrudescence is poorly understood. Therefore, the aim of the present study was to determine the role of the stress hormone cortisol in *Salmonella *recrudescence by pigs. We showed that a 24 h feed withdrawal increases the intestinal *Salmonella *Typhimurium load in pigs, which is correlated with increased serum cortisol levels. A second in vivo trial demonstrated that stress related recrudescence of *Salmonella *Typhimurium in pigs can be induced by intramuscular injection of dexamethasone. Furthermore, we found that cortisol, but not epinephrine, norepinephrine and dopamine, promotes intracellular proliferation of *Salmonella *Typhimurium in primary porcine alveolar macrophages, but not in intestinal epithelial cells and a transformed cell line of porcine alveolar macrophages. A microarray based transcriptomic analysis revealed that cortisol did not directly affect the growth or the gene expression or *Salmonella *Typhimurium in a rich medium, which implies that the enhanced intracellular proliferation of the bacterium is probably caused by an indirect effect through the cell. These results highlight the role of cortisol in the recrudescence of *Salmonella *Typhimurium by pigs and they provide new evidence for the role of microbial endocrinology in host-pathogen interactions.

## Introduction

For a long time it has been known that stress may cause recrudescence of some bacterial infections in food-producing animals, such as poultry and pigs [1,2]. Salmonellosis is one of the most important zoonotic bacterial diseases and pigs are considered as one of the main sources of human salmonellosis [3-6]. Worldwide, *Salmonella enterica *subspecies *enterica *serovar Typhimurium (*Salmonella *Typhimurium) is the predominant serovar isolated from slaughter pigs [7]. Pigs infected with *Salmonella *Typhimurium can carry this bacterium asymptomatically in their tonsils, gut and gut-associated lymphoid tissue for months resulting in so called *Salmonella *carriers. Generally, these persistently infected animals intermittently shed low numbers of *Salmonella *bacteria. However, during periods of stress, like transport to the slaughter house, recrudescence of *Salmonella *may occur. This results in increased cross-contamination during transport and lairage and to a higher degree of carcass contamination, which could lead to higher numbers of foodborne *Salmonella *infections in humans [4,8]. Until now, the mechanism of stress related recrudescence of *Salmonella *is not well understood and this study aimed at elucidating this phenomenon.

Although stress is hard to define and the factors causing stress can be very different, they generally result in similar physiological responses. A period of stress results in the release of a variety of neurotransmitters, peptides, cytokines, hormones, and other factors into the circulation or tissues of the stressed organism [9-11]. Besides the fast-acting catecholamines, which are released by the sympathetic nervous system, the hypothalamic-pituitary-adrenal axis becomes activated, resulting in the release of the slow-acting glucocorticoids by the adrenal gland [12]. These stress hormones can not only affect the host immune response via the modulation of various aspects of the immune system, but they also can have a direct effect on the bacteria and may influence their interactions with the host cells [13]. Indeed, several bacterial species can exploit the neuroendocrine alteration of a host stress reaction as a signal for growth and pathogenic processes [12,14,15].

Pigs secrete cortisol as the predominant glucocorticoid [16]. Therefore, it was the aim of the present study to determine the role of this hormone in the stress related recrudescence of *Salmonella *Typhimurium by pigs and to elucidate if it alters bacterium-host cell interactions.

## Materials and methods

### Chemicals

Cortisol and dexamethasone (Sigma-Aldrich, Steinheim, Germany) stock solutions of 10 mM were prepared in water and stored at - 20°C. Serial dilutions of cortisol were, depending on the experiment, prepared in Luria-Bertani broth (LB, Sigma-Aldrich NV/SA) or in the corresponding cell culture medium.

### Bacterial strains and growth conditions

*Salmonella *Typhimurium strain 112910a, isolated from a pig stool sample and characterized previously by Boyen et al., was used as the wild type strain in which the spontaneous nalidixic acid resistant derivative strain (WT_nal_) was constructed [17]. For fluorescence microscopy, *Salmonella *Typhimurium strain 112910a carrying the pFPV25.1 plasmid expressing green fluorescent protein (GFP) under the constitutive promoter of *rpsM *was used [17,18].

Unless otherwise stated, the bacteria were generally grown overnight (16 to 20 h) as a stationary phase culture with aeration at 37°C in 5 mL of LB broth. To obtain highly invasive late logarithmic cultures for invasion assays, 2 μL of a stationary phase culture were inoculated in 5 mL LB broth and grown for 5 h at 37°C without aeration [19].

For the oral inoculation of pigs, the WT_nal _was used to minimize irrelevant bacterial growth when plating tonsillar, lymphoid, intestinal and faecal samples. The bacteria were grown for 16 h at 37°C in 5 mL LB broth on a shaker, washed twice in Hank's buffered salt solution (HBSS, Gibco, Life Technologies, Paisley, Scotland) by centrifugation at 2 300 × *g *for 10 min at 4°C and finally diluted in HBSS to the appropriate concentration of 10^7 ^colony forming units (CFU) per mL. The number of viable *Salmonella *bacteria per mL inoculum was determined by plating 10-fold dilutions on Brilliant Green agar (BGA, international medical products, Brussels, Belgium) supplemented with 20 μg/mL nalidixic acid (BGA^NAL^, Sigma-Aldrich) for selective growth of the mutant strains.

### Cell cultures

Primary porcine alveolar macrophages (PAM) were isolated by broncho-alveolar washes from lungs of euthanized 3 to 4 week old piglets, obtained from a *Salmonella*-negative farm, as described previously [20]. The isolated cells were pooled and frozen in liquid nitrogen until further use. Prior to seeding the cells, frozen aliquots of approximately 10^8 ^cells/mL were thawed and washed 3 times in Hank's buffered salt solution with Ca^2+ ^and Mg^2+ ^(HBSS+, Gibco) with 10% (v/v) fetal calf serum (FCS, Hyclone, Cramlington, England) at 4°C. Finally, these cells were cultured in Roswell Park Memorial Institute medium (RPMI, Gibco) containing 10% (v/v) FCS, 2 mM L-glutamine (Gibco), 1 mM sodium pyruvate (Gibco), 1% (v/v) non essential amino acids (NEAA, Gibco), 100 units penicillin per mL and 100 μg streptomycin per mL (penicillin-streptomycin, Gibco). The porcine macrophage cell line (3D4/31) is derived from PAM and was obtained from Weingartl et al. [21]. These cells were grown in Dulbecco's modified Eagle's medium (DMEM, Gibco) supplemented with 1% (v/v) NEAA and 10% (v/v) FCS.

The polarized intestinal porcine epithelial (IPEC-J2) cell line is derived from jejunal epithelia isolated from a neonatal piglet and was grown in DMEM supplemented with 47% (v/v) Ham's F12 medium (Gibco), 5% (v/v) FCS, 1% insulin-transferrin-selenium-A supplement (ITS, Gibco), and antibiotics as described above [22,23].

### In vivo trials

All animal experiments were carried out in strict accordance with the recommendations in the European Convention for the Protection of Vertebrate Animals used for Experimental and other Scientific Purposes. The protocols were approved by the ethical committee of the Faculty of Veterinary Medicine, Ghent University (EC 2007/101 and EC 2010/108).

#### Experimental inoculation of piglets

A standardized infection model was used to create *Salmonella *carrier pigs [24]. For this purpose, four-week-old piglets (commercial closed line based on Landrace) were obtained from a serologically negative breeding herd (according to the Belgian *Salmonella *monitoring program). The *Salmonella*-free status of the piglets was verified serologically using a commercially available *Salmonella *antibody test kit (IDEXX, Hoofddorp, The Netherlands), and bacteriologically via repeated faecal sampling. The piglets were housed in pairs in separate isolation units at 25°C under natural day-night rhythm with *ad libitum *access to feed and water. Seven days after they arrived, the piglets were orally inoculated with 2 mL HBSS containing 10^7 ^CFU of WT_nal _per mL.

In a first in vivo trial (EC 2007/101), we investigated the effect of different types of stress on the recrudescence of *Salmonella *Typhimurium by pigs. In a second in vivo trial, (EC 2010/108), we injected pigs intramuscularly with 2 mg dexamethasone per kg body weight to test our hypothesis that corticosteroids induce the recrudescence of *Salmonella *Typhimurium in pigs.

#### Effect of different types of stress on the *Salmonella *Typhimurium load in carrier pigs

At day 23 post inoculation (pi), pigs were submitted to either social stress (*n *= 12) or feed withdrawal stress (*n *= 6), mimicking the transport and starvation period before slaughter, respectively. The remaining six pigs were not stressed and served as a negative control group. To induce social stress, the piglets were mixed for 24 h. One piglet was removed from its pen and transferred to another pen, which already contained 2 piglets. This was done in triplicate, so finally there were three groups of 3 piglets per pen (overcrowding) and three groups of 1 piglet per pen (isolation). To mimic feed withdrawal stress, three groups of 2 piglets per pen were not fed for 24 h. After the stress period, the animals were euthanized. Blood samples were taken of all pigs at the same time and the serum cortisol concentrations were determined in twofold via a commercially available enzyme-linked immunosorbent assay (ELISA, Neogen, Lansing, USA), according to the manufacterer's instructions. Furthermore, samples of tonsils, ileocaecal lymph nodes, ileum, caecum, colon and contents of ileum, caecum and colon were collected for bacteriological analysis to determine the number of *Salmonella *bacteria, with a detection limit of 50 CFU per gram tissue or contents.

#### Effect of dexamethasone on the *Salmonella *Typhimurium load in carrier pigs

The animals (*n *= 18) were housed and inoculated as described above to create *Salmonella *carrier pigs [17]. At day 42 pi, pigs were intramuscularly injected with either dexamethasone (Kela laboratoria, Hoogstraten, Belgium) (*n *= 9) or HBSS (*n *= 9). Since cortisol has a short half-life of 1 to 2 h [25], we used dexamethasone, which is a long-acting glucocorticoid with a half-life of 36 to 72 h [26], at a concentration of 2 mg dexamethasone per kg body weight. Pigs are remarkably resistant to dexamethasone-mediated immunosuppression at the dose used [27]. At 24 h after dexamethasone injection, the animals were euthanized and samples of tonsils, ileocaecal lymph nodes, ileum, caecum, colon and contents of ileum, caecum and colon were collected for bacteriological analysis, with a detection limit of 83 CFU per gram tissue or contents.

#### Bacteriological analysis

All tissues and samples were weighed and 10% (w/v) suspensions were prepared in buffered peptone water (BPW, Oxoid, Basingstoke, United Kingdom). The samples were homogenized with a Colworth stomacher 400 (Seward and House, London, United Kingdom) and the number of *Salmonella *bacteria was determined by plating 10-fold dilutions on BGA^NAL ^plates. These were incubated for 16 h at 37°C. The samples were pre-enriched for 16 h in BPW at 37°C and, if negative at direct plating, enriched for 16 h at 37°C in tetrathionate broth (Merck KGaA, Darmstadt, Germany) and plated again on BGA^NAL^. Samples that were negative after direct plating but positive after enrichment were presumed to contain 50 or 83 CFU per gram tissue or contents (detection limit for direct plating). Samples that remained negative after enrichment were presumed to contain less than 50 or 83 CFU per gram tissue or contents and were assigned value "1" prior to log transformation. Subsequently, the number of CFU for all samples derived from all piglets was converted logarithmically prior to calculation of the average differences between the log_10 _values of the different groups and prior to statistical analysis.

### The effects of cortisol and dexamethasone on host-pathogen interactions of *Salmonella *Typhimurium with porcine host cells

To examine whether the ability of *Salmonella *Typhimurium to invade and proliferate in primary porcine alveolar macrophages (PAM) and IPEC-J2 cells was altered after exposure of these cells to cortisol, invasion and intracellular survival assays were performed. For the invasion assays, PAM and IPEC-J2 cells were seeded in 24-well plates at a density of approximately 5 × 10^5 ^and 10^5 ^cells per well, respectively. PAM were allowed to attach for 2 h and IPEC-J2 cells were allowed to grow for 24 h. Subsequently, the cells were exposed to different concentrations of cortisol ranging from 0.001 to 100 μM. After 24 h, the invasion assay was performed as described by Boyen et al. [24]. Briefly, *Salmonella *was inoculated into the wells at a multiplicity of infection (MOI) of 10:1. To synchronize the infection, the inoculated multiwell plates were centrifuged at 365 × *g *for 10 min and incubated for 30 min at 37°C under 5% CO_2_. Subsequently, the cells were washed 3 times with HBSS+ and fresh medium supplemented with 100 μg/mL gentamicin (Gibco) was added. After 1 h incubation, the PAM and IPEC-J2 cells were washed 3 times and lysed for 10 min with 1% (v/v) Triton X-100 (Sigma-Aldrich) or 0.2% (w/v) sodium deoxycholate (Sigma-Aldrich), respectively, and 10-fold dilutions were plated on BGA plates.

To assess intracellular proliferation, cells were seeded and inoculated with *Salmonella *Typhimurium, but the medium containing 100 μg/mL gentamicin was replaced after 1 h incubation with fresh medium containing 20 μg/mL gentamicin, with or without cortisol or dexamethasone ranging from 0.001 to 100 μM. The number of viable bacteria was assessed 24 h after infection. To examine whether cortisol could also increase the intracellular proliferation of *Salmonella *Typhimurium in a macrophage cell line, the intracellular proliferation assay was repeated in the 3D14/31 cell line.

To determine whether the observed effect was cortisol specific, invasion and proliferation assays were performed also after exposure of PAM to epinephrine, norepinephrine or dopamine at concentrations ranging from 5 to 50 μM to reflect experiments previously performed by others [28].

To visualize the effect of cortisol on the intracellular proliferation of *Salmonella *bacteria, PAM were seeded in sterile Lab-tek^® ^chambered coverglasses (VWR, Leuven, Belgium), inoculated with GFP-producing *Salmonella *at a multiplicity of infection of 2:1, as described by Boyen et al. [24], and exposed to cortisol at a high physiological stress concentration of 1 μM [29] in cell medium or to cell medium only. After 24 h at 37°C, cells were washed three times to remove unbound bacteria and cellTrace™ calcein red-orange (Molecular Probes Europe, Leiden, The Netherlands) was added for 30 min at 37°C. Afterwards, cells were washed three times and fluorescence microscopy was carried out. Per experiment the number of cell associated bacteria was determined in 100 macrophages and the average number of cell associated bacteria was calculated from four independent experiments.

### The effect of cortisol on the viability of porcine host cells

It is possible that cortisol affects the toxicity of *Salmonella *Typhimurium for host cells, resulting in an increased or reduced cell death. Therefore, the cytotoxic effect of cortisol on uninfected and infected PAM and IPEC-J2 cells was determined using the neutral red uptake assay. For this purpose, PAM were seeded in a 96-well microplate at a density of approximately 2 × 10^5 ^cells per well and were allowed to attach for 2 h. The IPEC-J2 cells were seeded and allowed to grow for 24 h in a 96-well microplate at a density of approximately 2 × 10^4 ^cells per well. As earlier, uninfected and infected cells with *Salmonella *Typhimurium were treated with medium whether or not supplemented with cortisol concentrations ranging from 0.001 to 100 μM for 24 h. To assess cytotoxicity, 150 μL of freshly prepared neutral red solution (33 μg/mL in DMEM without phenol red) prewarmed to 37°C was added to each well and the plate was incubated at 37°C for an additional 2 h. The cells were then washed two times with HBSS+ and 150 μL of extracting solution ethanol/Milli-Q water/acetic acid, 50/49/1 (v/v/v), was added in each well. The plate was shaken for 10 min. The absorbance was determined at 540 nm using a microplate ELISA reader (Multiscan MS, Thermo Labsystems, Helsinki, Finland). The percentage of viable cells was calculated using the following formula:

% cytotoxicity=100 × ((a−b)/(c−b))

In this formula a = OD_540 _derived from the wells incubated with cortisol, b = OD_540 _derived from blank wells, c = OD_540 _derived from untreated control wells.

### Effect of cortisol on the growth and gene expression of *Salmonella *Typhimurium

#### Effect of cortisol on the growth of *Salmonella *Typhimurium

It is possible that cortisol directly increases the growth of *Salmonella *Typhimurium. Therefore, we examined the effect of cortisol concentrations (1 nM, 100 nM, 1 μM and 100 μM) on the growth of *Salmonella *Typhimurium, during 24 h. For this purpose, *Salmonella *Typhimurium was grown in LB broth or DMEM medium with or without cortisol. The number of CFU per mL was determined at different time points (*t *= 0, 2.5, 5, 7.5 and 24 h) by titration of 10-fold dilutions of the bacterial suspensions on BGA. After incubation for 24 h at 37°C, the number of colonies was determined.

#### Effect of cortisol on the gene expression of *Salmonella *Typhimurium

A direct effect of cortisol on the pathogenicity of *Salmonella *Typhimurium could explain the increased intracellular proliferation in macrophages. Therefore, a microarray analysis was conducted to investigate the effect of cortisol on the gene expression of the bacterium.

RNA was isolated from *Salmonella *Typhimurium at logarithmic and stationary growth phase (2.00 OD_600 nm _units) in the presence or absence of 1 μM cortisol, for 5 and 16 h respectively [19]. This was done according to procedures described on the IFR microarray web site [30]. The quantity and purity of the isolated RNA was determined using a Nanodrop spectrophotometer and Experion RNA StdSens Analysis kit (Biorad).

Triple biological replicates were performed for each experiment, RNA labeled and hybridized to *Salmonella *Typhimurium SALSA2 microarrays, consisting of 5080 ORFs, according to protocols described on the IFR microarray web site [30]. Following washing and scanning of the hybridized microarrays, the expression data was processed and statistically filtered. All transcriptomic data was normalized to that of the wild-type strain. A Benjamini and Hochberg multiple testing correction was then applied to adjust individual *P*-values so that only data with a false discovery rate of 0.05 and a ≥ 1.5-fold change in the expression level was retained.

The microarray data discussed in this publication are MIAME compliant and have been deposited in NCBI's Gene Expression Omnibus [31] and are accessible through GEO Series accession number GSE30923 [32].

### Statistical analysis

All in vitro experiments were conducted in triplicate with 3 repeats per experiment, unless otherwise stated. All statistical analyses were performed using SPSS version 17 (SPSS Inc., Chicago, IL, USA). Normally distributed data were analyzed using unpaired Student's *t*-test or one-way ANOVA to address the significance of difference between mean values with significance set at *p *≤ 0.05. Bonferroni as post hoc test was used when equal variances were assessed. If equal variances were not assessed, the data were analyzed using Dunnett's T3 test. Not normally distributed data were analyzed using the non parametric Kruskal-Wallis analysis, followed by Mann-Whitney U test.

## Results

### Feed withdrawal results in increased numbers of *Salmonella *Typhimurium bacteria in the gut of pigs and elevated blood cortisol levels

**Figure 1:**
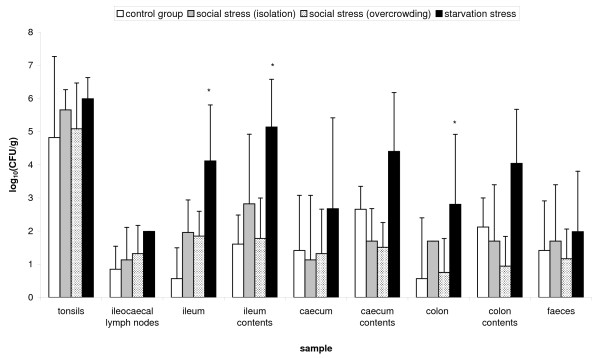
**Effect of different types of stress on the *Salmonella *load in carrier pigs**. Recovery of *Salmonella *Typhimurium bacteria from various organs and gut contents of carrier pigs that were subjected to either feed withdrawal (*n *= 6) or social stress, isolation (*n *= 3) and overcrowding (*n *= 9), 24 h before euthanasia. Six pigs were not stressed and served as a control group. The log_10 _value of the ratio of CFU/gram sample is given as the mean + standard deviation. Superscript (*) refers to a significant difference compared to the control group (*p *≤ 0.05).

Carrier pigs subjected to feed withdrawal, 24 h before euthanasia, showed elevated numbers of *Salmonella *Typhimurium per gram in their bowel contents and organs in comparison to the control group (Figure 1). This increase was significant in the ileum (*p *≤ 0.001), ileum contents (*p *= 0.022) and colon (*p *= 0.014). The social stress groups (overcrowding and isolation) showed no significant differences in comparison to the control group.

Pigs that were subjected to feed withdrawal (*p *= 0.004) and overcrowding (*p *= 0.001) showed significantly elevated serum cortisol levels compared to the control group that had a mean cortisol concentration ± standard deviation of 48.65 ± 4.67 nM. Pigs that were starved 24 hours before euthanasia had the highest mean serum cortisol level ± standard deviation of 66.88 ± 6.72 nM. Pigs that were housed per 3 (overcrowding) and housed separately (isolation) at 24 h before euthanasia, had a mean cortisol concentration ± standard deviation of 59.26 ± 3.47 nM and 53.66 ± 2.06 nM, respectively.

### Dexamethasone increases the number of *Salmonella *Typhimurium bacteria in the gut of carrier pigs

**Figure 2:**
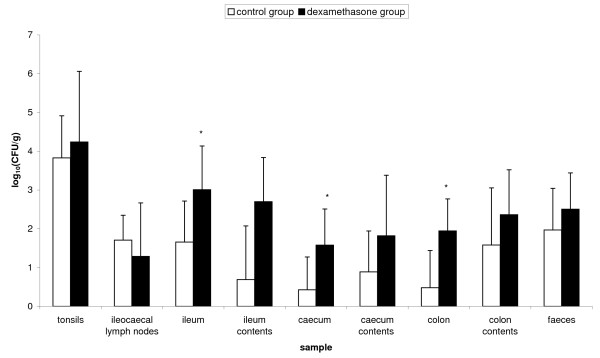
**Effect of dexamethasone on the *Salmonella *Typhimurium load in carrier pigs**. Recovery of *Salmonella *Typhimurium bacteria from various organs and gut contents of carrier pigs that were injected with either HBSS (control group, *n *= 9) or 2 mg dexamethasone per kg body weight (dexamethasone group, *n *= 9), 24 h before euthanasia. The log_10 _value of the ratio of CFU/gram sample is given as the mean + standard deviation. Superscript (*) refers to a significant difference compared to the control group (*p *≤ 0.05).

Carrier pigs that were intramuscularly injected with 2 mg dexamethasone per kg body weight, 24 h before euthanasia, showed elevated numbers of *Salmonella *Typhimurium in their gut tissues and contents in comparison to the control group that was intramuscularly injected with HBSS (Figure 2). This increase was significant in the ileum (*p *= 0.018), caecum (*p *= 0.014) and colon (*p *= 0.003).

### Cortisol and dexamethasone, but not catecholamines, promote the intracellular proliferation of *Salmonella *Typhimurium in primary porcine macrophages but not in 3D4/31 and IPEC-J2 cells

**Figure 3:**
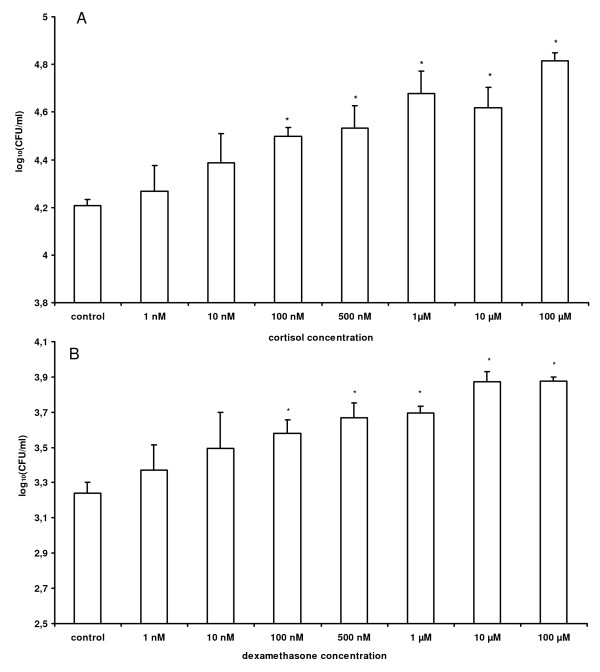
**Effect of cortisol and dexamethasone on the intracellular proliferation of *Salmonella *Typhimurium in macrophages**. Number of intracellular *Salmonella *Typhimurium bacteria in PAM that were treated with control medium or different concentrations of A) cortisol or B) dexamethasone, for 24 h after invasion. The log_10 _values of the number of gentamicin protected bacteria + standard deviation are shown. Results are presented as a representative experiment conducted in triplicate. Superscript (*) refers to a significant difference compared to the control (*p *≤ 0.05).

The results of the intracellular survival assay of *Salmonella *Typhimurium in PAM with or without exposure to cortisol or dexamethasone are summarized in Figure 3. Exposure to concentrations (> 100 nM) of cortisol or dexamethasone for 24 h led to a significant dose-dependent increase of the number of intracellular *Salmonella *Typhimurium bacteria compared to non-treated PAM.

Cortisol concentrations ranging from 0.001 to 100 μM did neither affect the intracellular proliferation of *Salmonella *Typhimurium in IPEC-J2 and 3D4/31 cells (Additional file 1), nor the invasion in PAM and IPEC-J2 cells (Additional file 2).

The enhanced intracellular proliferation of *Salmonella *Typhimurium in PAM exposed to a high physiological stress concentration of 1 μM cortisol [29] was confirmed in a proliferation assay with GFP-*Salmonella*. No difference was seen in the mean number of macrophages containing GFP-*Salmonella *± standard error of the mean, after exposure to 1 μM cortisol for 24 h in comparison to untreated PAM (41.0 ± 0.53 versus 40.5 ± 0.59 percentage *Salmonella *positive macrophages, respectively). However, the proliferation rate of intracellular bacteria that were exposed to 1 μM cortisol for 24 h was significantly (*p *= 0.001) increased in comparison with the control PAM, resulting in a higher mean bacterial count ± standard error of the mean (3.1 ± 0.14 versus 2.0 ± 0.07 bacteria per macrophage, respectively).

Epinephrine, norepinephrine and dopamine at a concentration of 1 μM did neither affect the invasion nor the intracellular proliferation of *Salmonella *Typhimurium in PAM (Additional file 3) and IPEC-J2 cells (Additional file 4).

### Cortisol does not affect the viability of primary porcine macrophages and intestinal epithelial cells and it does not directly affect *Salmonella *cytotoxicity, growth and gene expression

**Figure 4:**
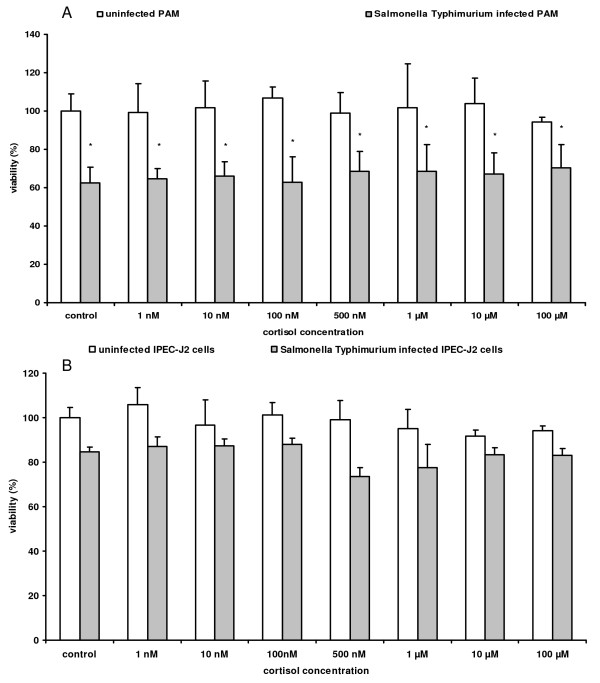
**Effect of cortisol on the viability of *Salmonella *infected and uninfected macrophages and IPEC-J2 cells**. Percentage viability (%) of *Salmonella *Typhimurium infected and uninfected (A) PAM and (B) IPEC-J2 cells, exposed to different concentrations of cortisol (0.001-100 μM). Twenty-four hours after incubation with cortisol, the cytotoxic effect was determined by neutral red assay. Results represent the means of three independent experiments conducted in triplicate and their standard deviation. Superscript (*) refers to a significant difference compared to control uninfected IPEC-J2 cells (*p *≤ 0.05).

Cortisol concentrations ranging from 0.001 to 100 μM did neither affect the viability of PAM and IPEC-J2 cells, nor the cytotoxicity of *Salmonella *Typhimurium for these cells (Figure 4). Furthermore, we showed that cortisol did not affect the growth of *Salmonella *Typhimurium in LB and DMEM medium (Additional file 5) and transcriptomic analysis revealed that exposure of stationary and logarithmic phase cultures of *Salmonella *Typhimurium to cortisol at a high physiological stress concentration of 1 μM [29], did not significantly affect gene expression levels compared to the untreated strain in LB medium (GSE30923).

## Discussion

Conflicting results have been published concerning the effect of different stressors on the shedding of *Salmonella *Typhimurium in pigs [33-38]. However, our findings elucidate that a natural stress stimulus like feed withdrawal causes recrudescence of a *Salmonella *Typhimurium infection in carrier pigs. Feed withdrawal before transport to the slaughterhouse is a common practice to reduce the risk of carcass and environmental contamination because a decrease of the gastrointestinal tract weight results in a lower risk of lacerations during evisceration [39]. However, we showed that feed withdrawal practices could result in an increased risk of contamination. Martín-Peláez et al. hypothesized that the increased faecal excretion of *Salmonella *Typhimurium after feed withdrawal could be the result of a lower short-chain fatty acids concentration, an increased pH or an increased number of lactic acid bacteria such as lactobacilli [34].

Until now, the mechanism of stress related recrudescence of *Salmonella *is not well understood and the investigation of this phenomenon is hindered by the lack of appropriate animal models [40,41]. The higher *Salmonella *Typhimurium numbers in pigs subjected to feed withdrawal stress, suggest that this model is a valuable tool for the study of stress related *Salmonella *recrudescence. We hypothesized that cortisol plays a role in the stress related recrudescence of *Salmonella *Typhimurium by pigs. During a stress reaction, the sympathetic nervous system and hypothalamic-pituitary-adrenal axis become activated, resulting in the release of catecholamines and glucocorticoids, respectively [15]. These stress hormones can affect the host immune response, but the pathogenesis of an infection can also be altered by direct effects of these stress mediators on the bacteria [13].

We showed that social stress and starvation result in elevated serum cortisol levels. Starvation can result in hypoglycaemia, which causes an increased secretion of cortisol to stimulate the gluconeogenesis [42]. Müller et al. showed that a starvation period up to 5 days in miniature pigs, results in a slight, but insignificant elevation of plasma cortisol levels [43]. Therefore, the elevated serum cortisol levels, seen in the carrier pigs that were subjected to feed withdrawal is probably the result of a combination between the feed withdrawal itself and the stress that is involved.

We revealed that a short-term treatment of carrier pigs with a high dose of dexamethasone results in the recrudescence of *Salmonella *Typhimurium. This confirms that the release of corticosteroids in the bloodstream itself could alter the outcome of a *Salmonella *Typhimurium infection in pigs, resulting in recrudescence of the infection. Smyth et al. showed that long-term treatment of mice with dexamethasone promotes a dose-dependent increase in *Salmonella *Typhimurium growth within mouse livers and spleens [44]. The increased numbers of bacteria described by Smyth et al. are probably the result of the immunosuppressive activity of glucocorticoids. Pigs are remarkably resistant to immunosuppression of dexamethasone, even at a high dose of 2 mg/kg body weight [27,45-47]. Therefore, the dexamethasone induced recrudescense of *Salmonella *Typhimurium in pigs is probably not the direct consequence of the immunosuppressive activity of dexamethasone.

We also demonstrated that this glucocorticoid mediated effect was not the result of a direct effect on the bacterium. Earlier research has shown that norepinephrine in vitro promotes the growth and the motility of *Salmonella enterica *[48,49]. However, we provide evidence that cortisol does not cause an increase in growth in LB and DMEM medium, or any significant changes in the gene expression of *Salmonella *Typhimurium when grown in a complex medium, at a physiological stress concentration of 1 μM [29]. In contrast to the absence of a direct effect on the bacterium, we showed that cortisol and dexamethasone promote intracellular proliferation of *Salmonella *Typhimurium in porcine macrophages, in a dose-dependent manner at concentrations (0.1 to 100 μM) that do not exert a notable effect on cell viability. Nevertheless, this increased survival was not observed 3D4/31 and IPEC-J2 cells. Although *Salmonella *is an extensively studied bacterium, still many questions remain about the intracellular environment of *Salmonella *within different host cells. After invasion, *Salmonella *resides within *Salmonella *containing vacuoles (SCV) which serves a unique intracellular compartment where it resides and eventually replicates. Maturation of the SCV has been studied in different cell types and these studies indicate that the SCV biogenesis may not be generalized [50]. Possibly, cortisol affects the SCV biogenesis in primary macrophages and not in other cell types, which results in an increased survival of the bacterium in these primary macrophages.

Although we showed that catecholamines did neither affect the intracellular proliferation nor the invasion of *Salmonella *Typhimurium in primary macrophages and IPEC-J2 cells, catecholamines have been shown to promote the growth and motility of *Salmonella *[48,49,51]. Concentrations of the catecholamines were not determined in the in vivo trial since they have a half-life of approximately 3 min and because their serum levels change in matter of seconds [52,53]. However, it is commonly known that a stress reaction also results in the release of catecholamines. Recently, Pullinger et al. demonstrated that the release of norepinephrine in pigs by administration of 6-hydroxydopamine, enhances the faecal extretion of *Salmonella *Typhimurium [54]. Therefore, it is possible that catecholamines and glucocorticoids act in a synergistic way to cause a sudden increase of *Salmonella *Typhimurium shedding in stressed animals. Since stress is very common in food producing animals and since these stress hormones and derivatives are frequently used in human and animal medicine, their effects need further examination [55,56]. The elucidation of the mechanisms through which stress and its hormones alter the susceptibility to an infection could help to improve the prevention and treatment of *Salmonella *Typhimurium infections in pigs, and as a consequence help to reduce the number of cases of human salmonellosis.

In conclusion, we showed that the glucocorticoid cortisol is involved in a stress induced recrudescence of *Salmonella *Typhimurium in carrier pigs. In addition to this, we pointed out that cortisol promotes the intracellular proliferation of *Salmonella *Typhimurium in porcine macrophages which is caused by an indirect effect through the cell.

## Competing interests

The authors declare that they have no competing interests.

## Authors' contributions

EV, AT and NS performed the microarray analysis. EV, AVP, KVD and BL performed the animal experiments. EV, FB, FH, SC and FP conceived the study, participated in its design and coordination. EV, FH and FP co-drafted the manuscript. All authors read and approved the final manuscript.

## Supplementary Material

Additional file 1 **Effect of cortisol on the intracellular proliferation of *Salmonella *Typhimurium in IPEC-J2 and 3D4/31 cells**. Number of intracellular *Salmonella *Typhimurium bacteria in (A) IPEC-J2 cells and (B) 3D4/31 cells, that were treated with control medium or cortisol (0.001 μM-100 μM), for 24 h after invasion. The log_10 _values of the number of gentamicin protected bacteria + standard deviation are given. Results are presented as a representative experiment conducted in triplicate. Click here for file

Additional file 2 **Effect of cortisol on the invasion of *Salmonella *Typhimurium in macrophages and IPEC-J2 cells**. The invasiveness of *Salmonella *Typhimurium in (A) PAM and (B) IPEC-J2 cells, whether or not exposed to cortisol (0.001-100 μM) is shown. The log_10 _values of the number of gentamicin protected bacteria + standard deviation are given. Results are presented as a representative experiment conducted in triplicate. Click here for file

Additional file 3 **Effects of catecholamines on the invasion and intracellular proliferation of *Salmonella *Typhimurium in macrophages**. The invasiveness (A) and the survival (B), 24 h after invasion, of *Salmonella *Typhimurium in PAM whether or not exposed to epinephrine, norepinephrine or dopamine (5-50 μM) is shown. The log_10 _values of the number of gentamicin protected bacteria + standard deviation are given. Results are presented as a representative experiment conducted in sixfold. Click here for file

Additional file 4 **Effect of catecholamines on the invasion and intracellular proliferation of *Salmonella *Typhimurium in IPEC-J2 cells**. The invasiveness (A) and the survival (B), 24 h after invasion, of *Salmonella *Typhimurium in IPEC-J2 cells whether or not exposed to epinephrine, norepinephrine or dopamine (5-50 μM) is shown. The log_10 _values of the number of gentamicin protected bacteria + standard deviation are given. Results are presented as a representative experiment conducted in sixfold. Click here for file

Additional file 5 **Effect of cortisol on the growth of *Salmonella *Typhimurium**. The log_10 _values of the CFU/mL + standard deviation are given at different time points (*t *= 0, 2.5, 5, 7.5, 24 h). *Salmonella *Typhimurium growth was examined in (A) LB and (B) DMEM medium, with or without cortisol (0.001-100 μM). Results are presented as a representative experiment conducted in triplicate. Click here for file
