# Supplementary figures and images for: Stress induced Salmonella Typhimurium recrudescence in pigs coincides with cortisol induced increased intracellular proliferation in macrophages
Source: Vet Res. 2011 Dec 7;42(1):118. doi: 10.1186/1297-9716-42-118 (PMC3256119; doi:10.1186/1297-9716-42-118)

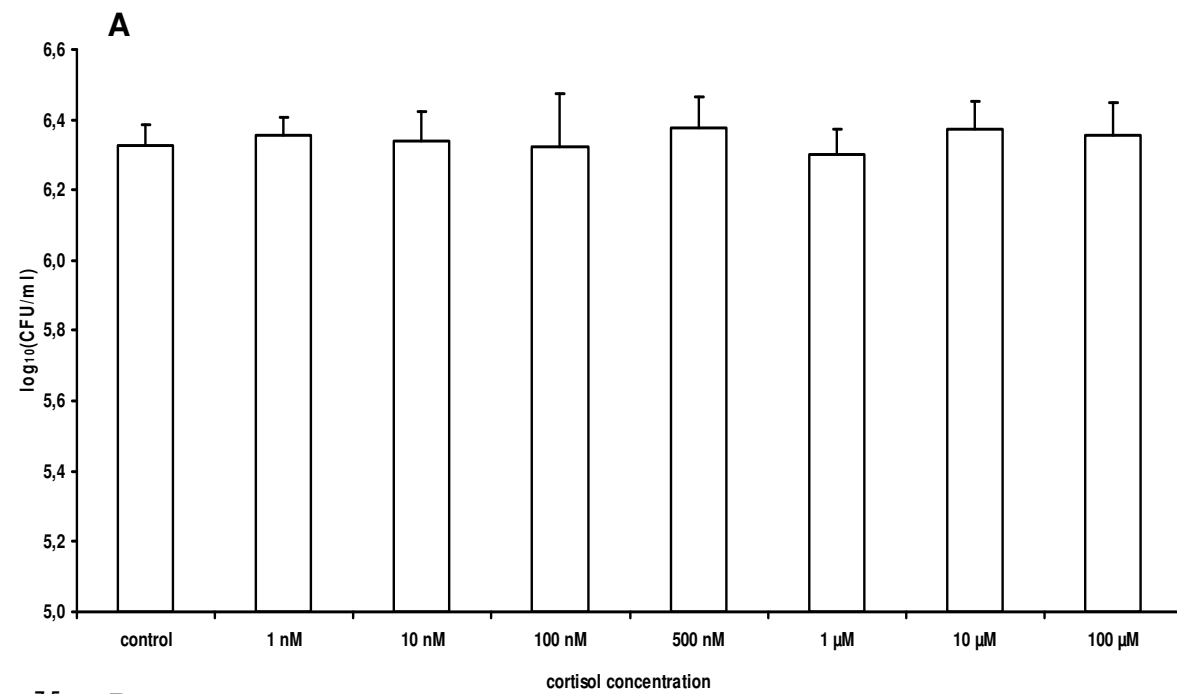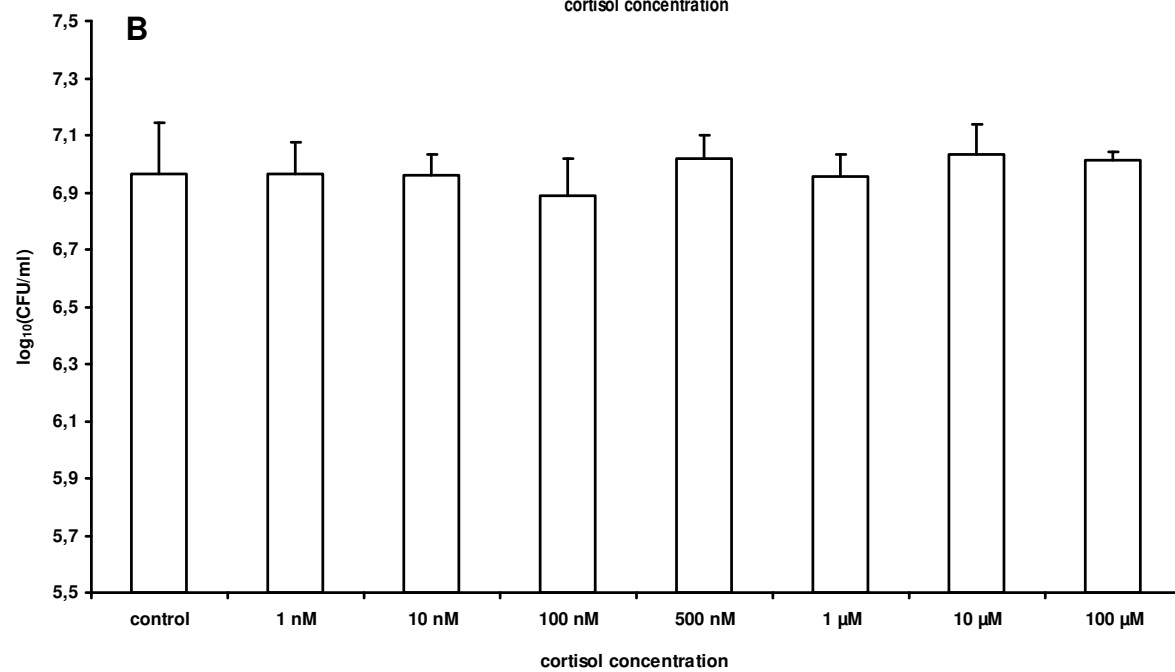

Supplement: Additional file 1 — Effect of cortisol on the intracellular proliferation of Salmonella Typhimurium in IPEC-J2 and 3D4/31 cells. Number of intracellular Salmonella Typhimurium bacteria in (A) IPEC-J2 cells and (B) 3D4/31 cells, that were treated with control medium or cortisol (0.001 μM-100 μM), for 24 h after invasion. The log10 values of the number of gentamicin protected bacteria + standard deviation are given. Results are presented as a representative experiment conducted in triplicate. [file 1297-9716-42-118-S1.PDF]

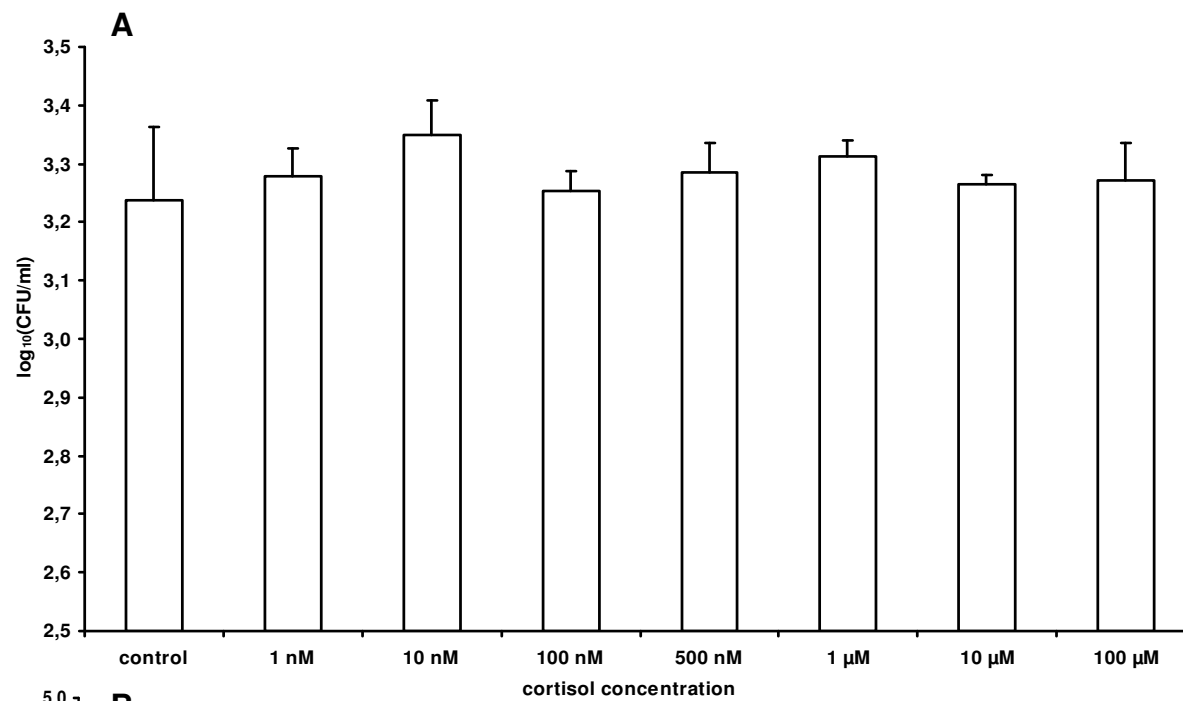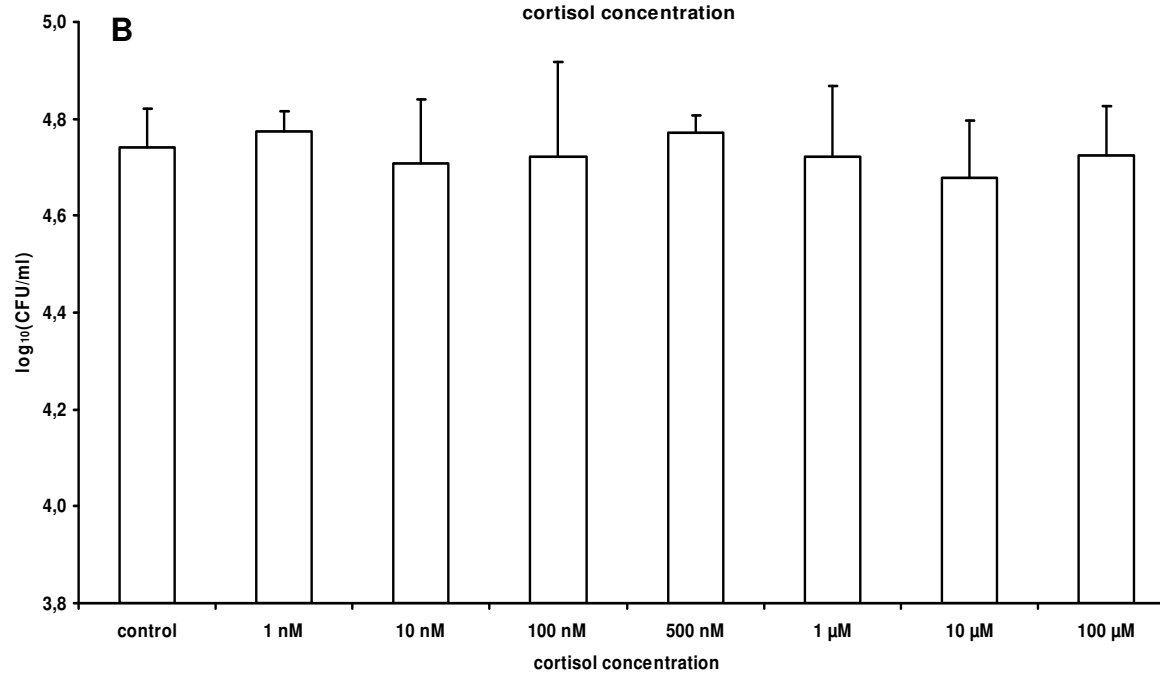

Supplement: Additional file 2 — Effect of cortisol on the invasion of Salmonella Typhimurium in macrophages and IPEC-J2 cells. The invasiveness of Salmonella Typhimurium in (A) PAM and (B) IPEC-J2 cells, whether or not exposed to cortisol (0.001-100 μM) is shown. The log10 values of the number of gentamicin protected bacteria + standard deviation are given. Results are presented as a representative experiment conducted in triplicate. [file 1297-9716-42-118-S2.PDF]

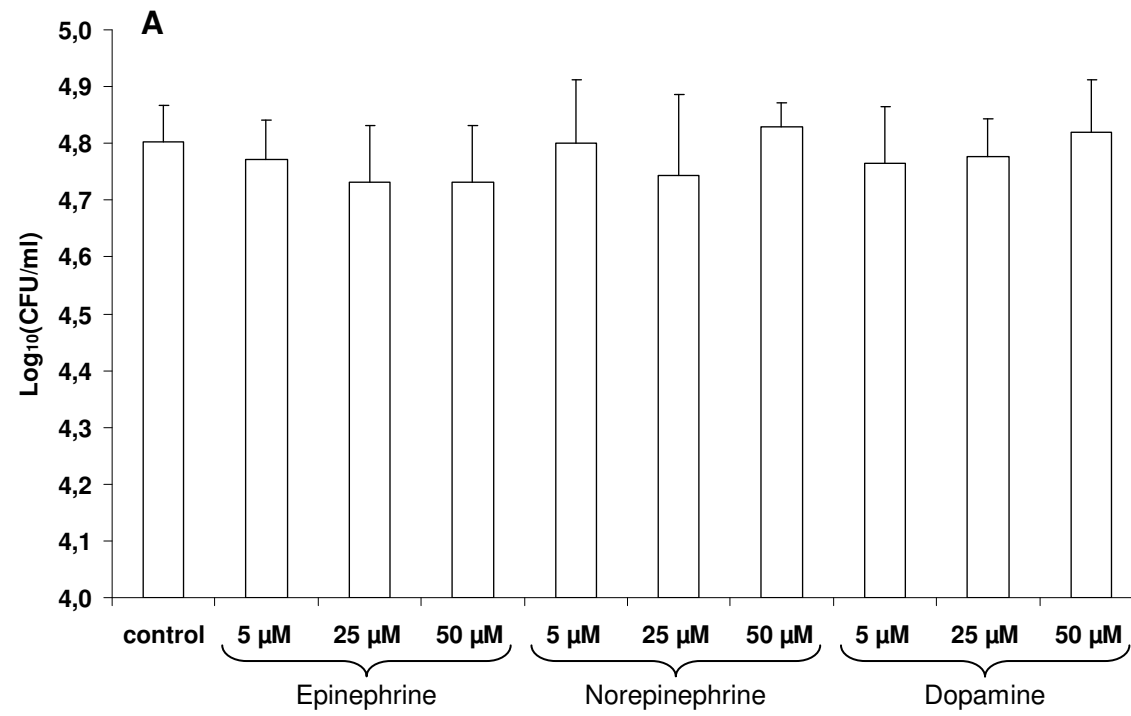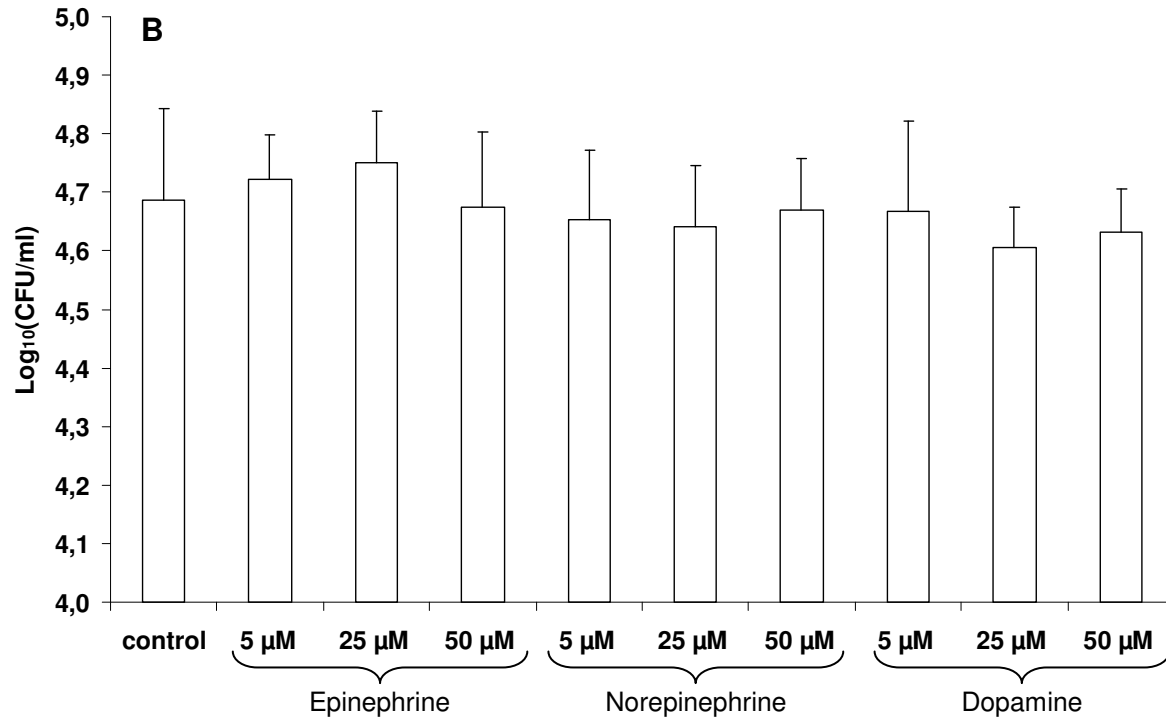

Supplement: Additional file 3 — Effects of catecholamines on the invasion and intracellular proliferation of Salmonella Typhimurium in macrophages. The invasiveness (A) and the survival (B), 24 h after invasion, of Salmonella Typhimurium in PAM whether or not exposed to epinephrine, norepinephrine or dopamine (5-50 μM) is shown. The log10 values of the number of gentamicin protected bacteria + standard deviation are given. Results are presented as a representative experiment conducted in sixfold. [file 1297-9716-42-118-S3.PDF]

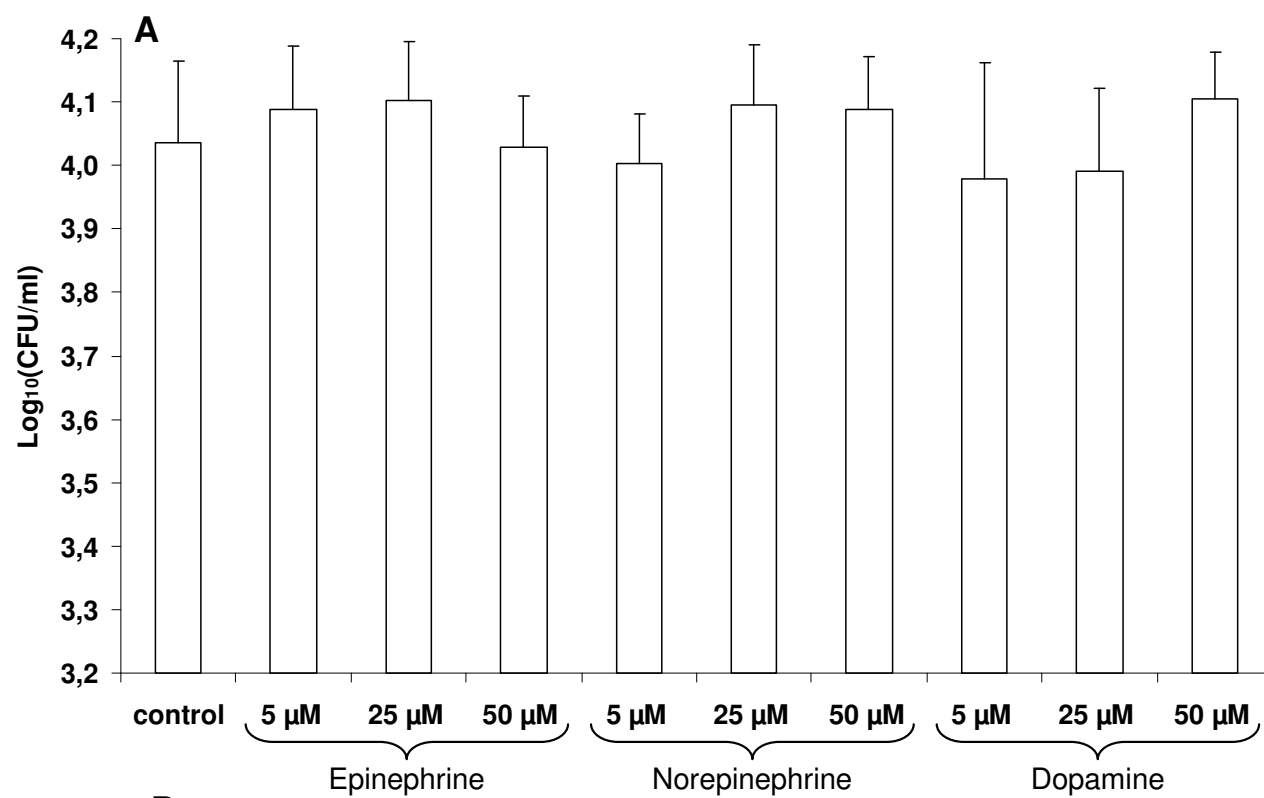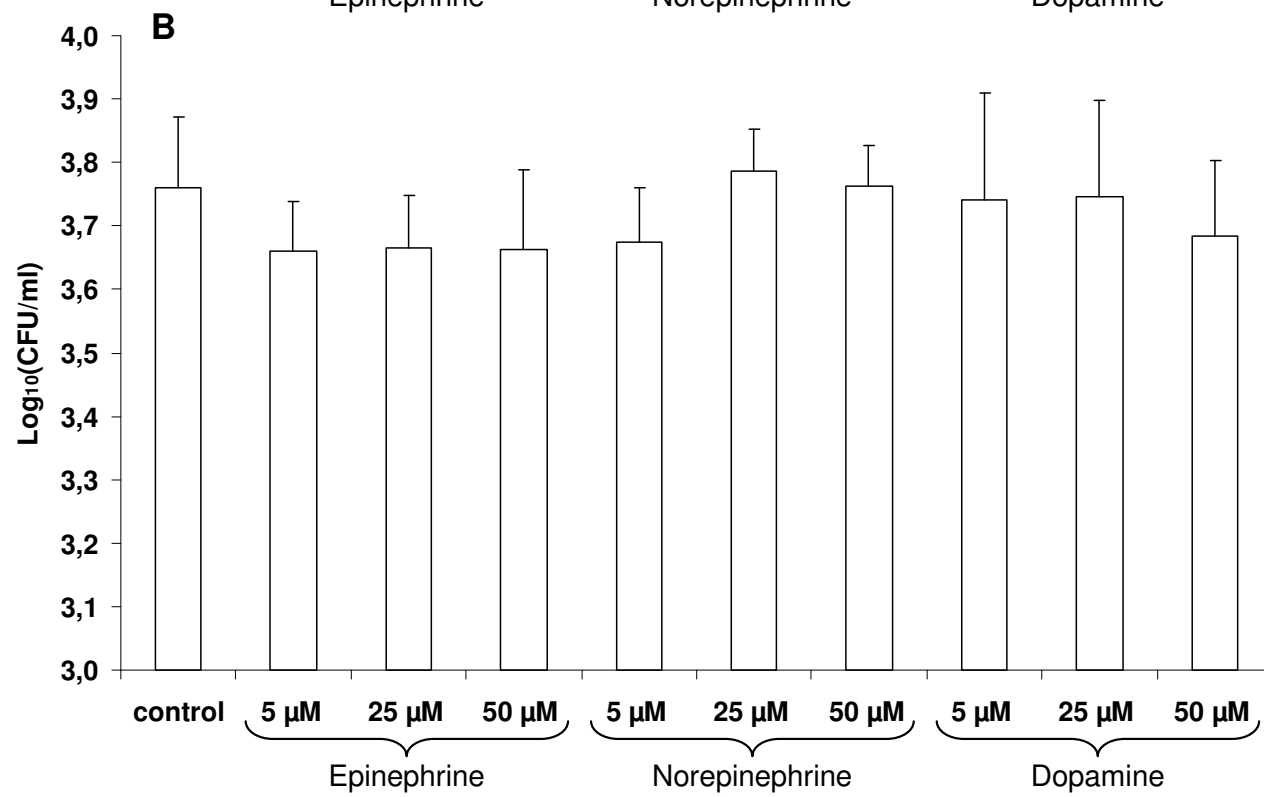

Supplement: Additional file 4 — Effect of catecholamines on the invasion and intracellular proliferation of Salmonella Typhimurium in IPEC-J2 cells. The invasiveness (A) and the survival (B), 24 h after invasion, of Salmonella Typhimurium in IPEC-J2 cells whether or not exposed to epinephrine, norepinephrine or dopamine (5-50 μM) is shown. The log10 values of the number of gentamicin protected bacteria + standard deviation are given. Results are presented as a representative experiment conducted in sixfold. [file 1297-9716-42-118-S4.PDF]

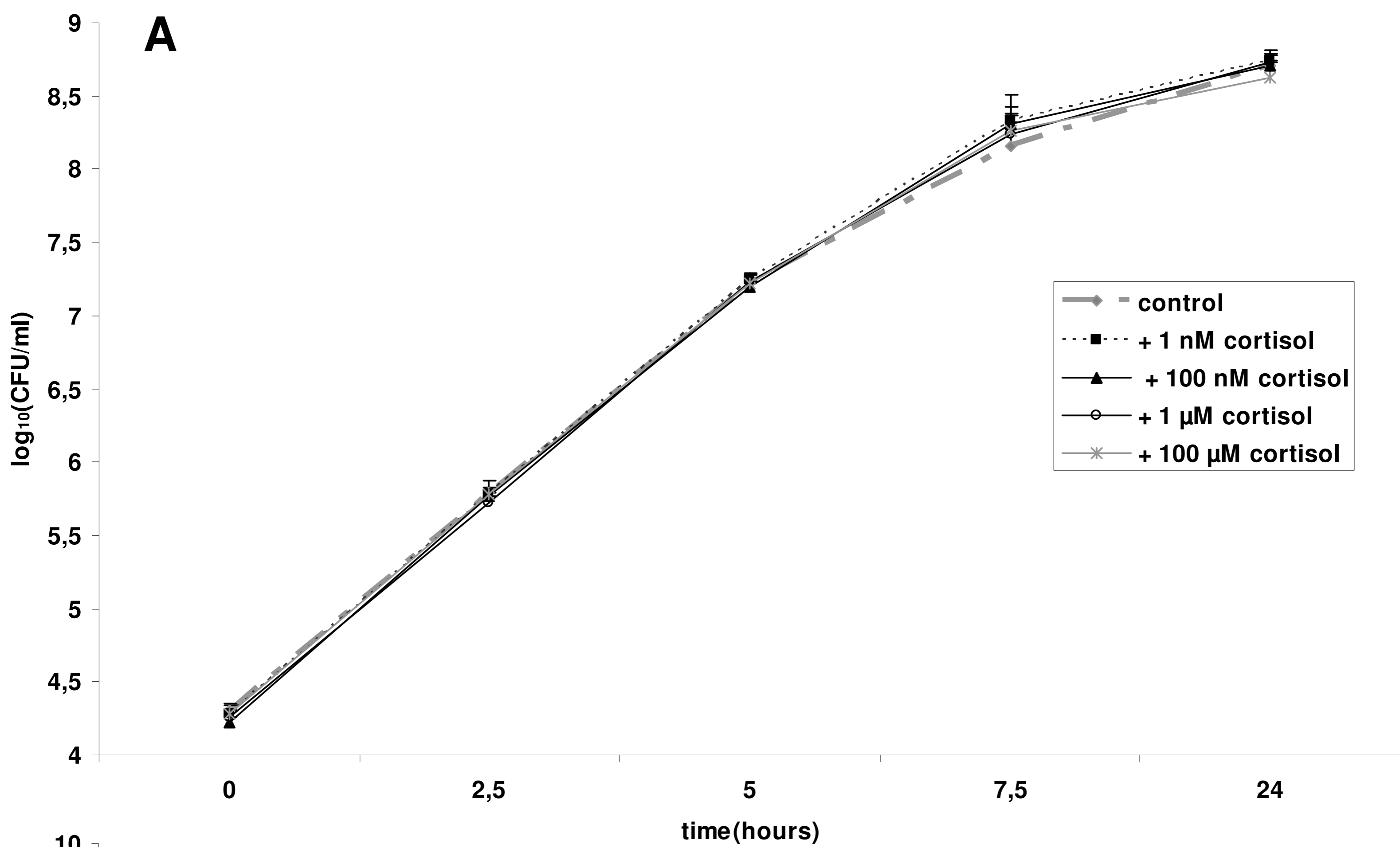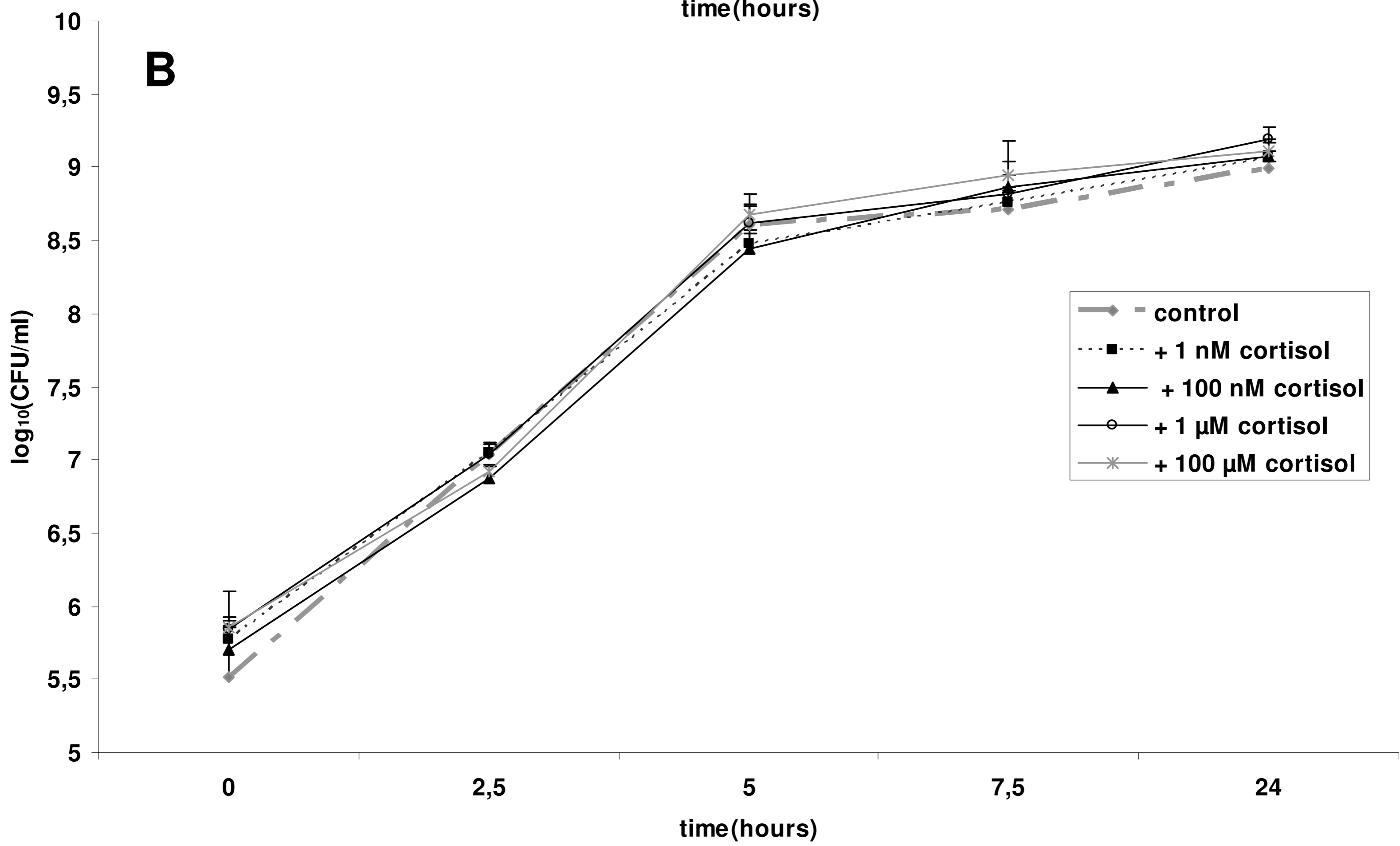

Supplement: Additional file 5 — Effect of cortisol on the growth of Salmonella Typhimurium. The log10 values of the CFU/mL + standard deviation are given at different time points (t = 0, 2.5, 5, 7.5, 24 h). Salmonella Typhimurium growth was examined in (A) LB and (B) DMEM medium, with or without cortisol (0.001-100 μM). Results are presented as a representative experiment conducted in triplicate. [file 1297-9716-42-118-S5.PDF]
